# Exercise intolerance in volume overload heart failure is associated with low carotid body mediated chemoreflex drive

**DOI:** 10.1038/s41598-021-93791-8

**Published:** 2021-07-14

**Authors:** David C. Andrade, Esteban Díaz-Jara, Camilo Toledo, Karla G. Schwarz, Katherin V. Pereyra, Hugo S. Díaz, Noah J. Marcus, Fernando C. Ortiz, Angélica P. Ríos-Gallardo, Domiziana Ortolani, Rodrigo Del Rio

**Affiliations:** 1 Laboratory of Cardiorespiratory Control, Department of Physiology, Pontificia Universidad Católica de Chile, Santiago, Chile; 2 Centro de Fisiología y Medicina de Altura, Facultad de Ciencias de la Salud, Universidad de Antofagasta, Antofagasta, Chile; 3 Dept. of Physiology and Pharmacology, Des Moines University, Des Moines, IA USA; 4 Mechanism of Myelin Formation and Repair Laboratory, Instituto de Ciencias Biomédicas, Facultad de Ciencias de Salud, Universidad Autónoma de Chile, Santiago, Chile; 5 Centro de Envejecimiento y Regeneración (CARE), Pontificia Universidad Católica de Chile, Santiago, Chile; 6 Centro de Excelencia en Biomedicina de Magallanes (CEBIMA), Universidad de Magallanes, Punta Arenas, Chile

**Keywords:** Cardiovascular diseases, Respiration

## Abstract

Mounting an appropriate ventilatory response to exercise is crucial to meeting metabolic demands, and abnormal ventilatory responses may contribute to exercise-intolerance (EX-inT) in heart failure (HF) patients. We sought to determine if abnormal ventilatory chemoreflex control contributes to EX-inT in volume-overload HF rats. Cardiac function, hypercapnic (HCVR) and hypoxic (HVR) ventilatory responses, and exercise tolerance were assessed at the end of a 6 week exercise training program. At the conclusion of the training program, exercise tolerant HF rats (HF + EX-T) exhibited improvements in cardiac systolic function and reductions in HCVR, sympathetic tone, and arrhythmias. In contrast, HF rats that were exercise intolerant (HF + EX-inT) exhibited worse diastolic dysfunction, and showed no improvements in cardiac systolic function, HCVR, sympathetic tone, or arrhythmias at the conclusion of the training program. In addition, HF + EX-inT rats had impaired HVR which was associated with increased arrhythmia susceptibility and mortality during hypoxic challenges (~ 60% survival). Finally, we observed that exercise tolerance in HF rats was related to carotid body (CB) function as CB ablation resulted in impaired exercise capacity in HF + EX-T rats. Our results indicate that: (i) exercise may have detrimental effects on cardiac function in HF-EX-inT, and (ii) loss of CB chemoreflex sensitivity contributes to EX-inT in HF.

## Introduction

Heart failure (HF) is a global public health problem characterized by autonomic abnormalities and impaired cardiac function^1–8^. Current pharmaceutical approaches to HF treatment are effective in delaying disease progression; however, the 5-year mortality rate is approximately 50%^9^. Exercise training (EX) has been shown to be an effective non-pharmacological therapeutic adjunct in treatment of HF^10–19^, that results in improvements in cardiac function, quality of life, and survival. These beneficial effects are frequently associated with improvement in cardiac autonomic imbalance and normalization of abnormal chemoreflex function^10–19^, both of which are associated with lower survival rates in HF^4,6^. However, these beneficial effects rely on the ability to tolerate EX, which is not always a given in patients with HF^20^.

Exercise intolerance (EX-inT) is defined as impairment of the ability to perform physical activity and is characterized by decreased exercise and functional capacities^21^. It is one of several important indicators in the diagnosis of patients with HF^22^. The precise mechanisms underlying EX-inT in HF are not fully understood; however, it has been proposed that EX-inT may be linked to reductions in perfusion of working muscles and consequent decreases in oxygen supply. Theoretically, this is a result of impaired ability to increase cardiac output to working muscles which is compounded by persistent sympathoexcitation and reduced vasodilatory mechanisms in vascular beds regulating muscle perfusion^23^. Most of the experimental support for this hypothesis comes from studies examining reduced ejection fraction HF, which is indeed, characterized by reductions in blood flow to muscle secondary to heart damage^24^. However, it is worth noting that patients with preserved ejection fraction HF, who often do not have hemodynamic compromise at rest also experience EX-inT.

In order to meet the metabolic demands of exercise, increases in ventilation are required in addition to increases in and re-distribution of cardiac output. Peripheral chemoreflexes are one of several important homeostatic mechanisms that contribute to increases in pulmonary ventilation during exercise. Early evidence of this, came from studies in patients who had undergone bilateral denervation of the peripheral chemoreceptors (i.e. carotid bodies) for treatment of bronchial asthma. In these people, carotid body resection resulted in a significant reduction in exercise hyperpnea^25^. Previous studies have shown that aberrant chemoreflex function contributes to autonomic dysfunction, abnormal breathing patterns, and cardiac dysfunction in HF^1,2,4–8^. However, to date no studies have comprehensively addressed the role of chemoreflex function in EX-inT in HF with preserved ejection fraction (HFpEF). Reductions in chemoreflex gain could potentially have an adverse impact on exercise tolerance in HF due to inadequate pulmonary ventilation during exercise. In the present study, we aimed to determine the prevalence of EX-inT in a volume-overload HF model (which lacks the confounding effect of reduced blood flow)^5,26^ and the extent to which aberrant chemoreflex function contributes to EX-inT in this model.

## Results

### Tolerance/intolerance to exercise training in HF animals

**Figure 1:**
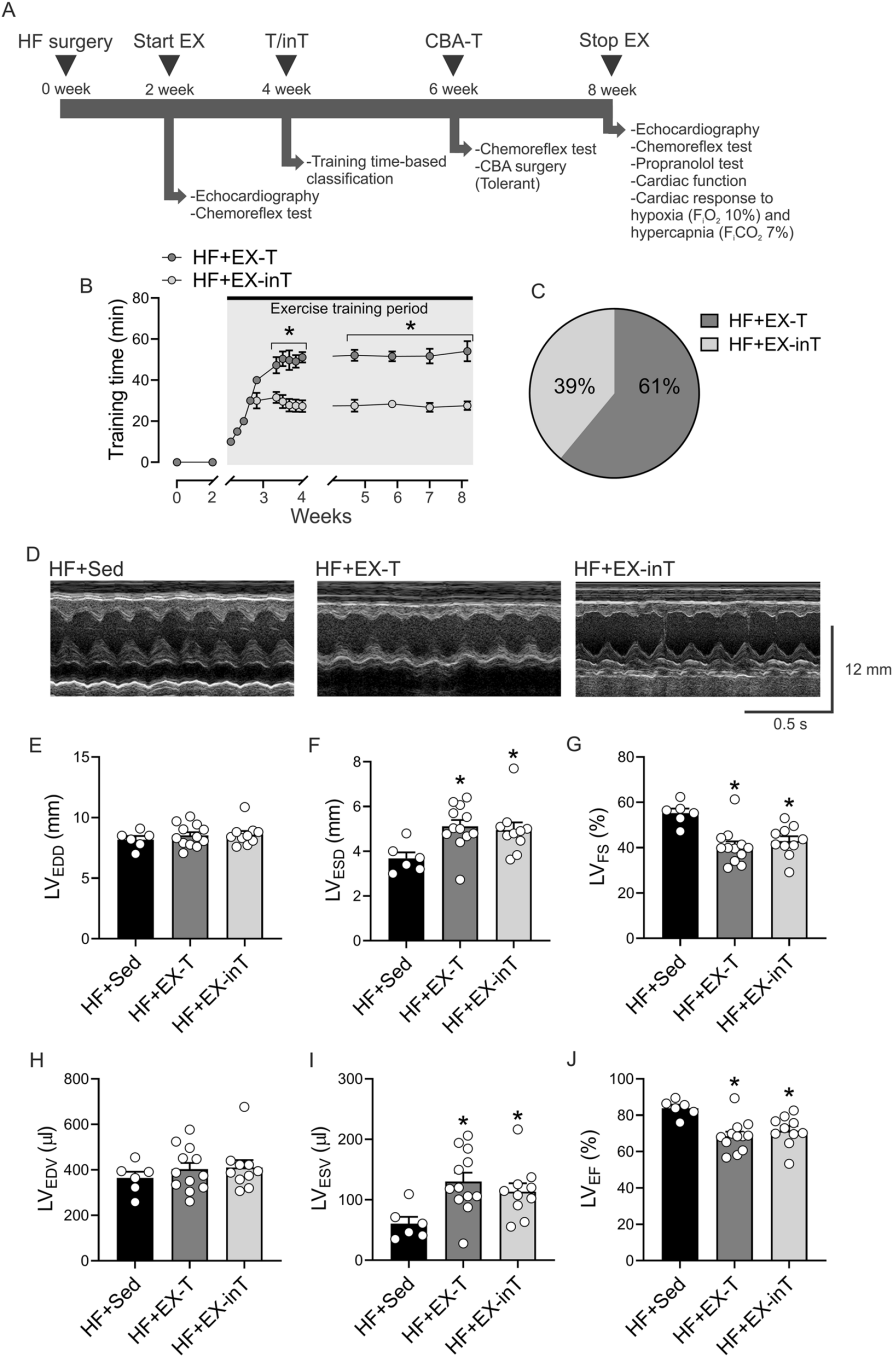
Prevalence of exercise tolerant (T) and intolerant (inT) heart failure (HF) rats and echocardiographic parameters after 6 weeks of exercise training (EX). (**A**) Timeline of experiments. HF surgery was performed at week 0. After 2 weeks, echocardiography and chemoreflex test were performed and then exercise training was started. Exercise tolerance (EX-T) and intolerance (EX-inT) classification was performed 2 weeks after the initiation of the exercise program. In a separate experiment, carotid bodies were ablated (CBA) bilaterally in HF + EX-T rats. Finally, at 8 weeks post-HF induction, we performed terminal physiological cardiorespiratory measurements. (**B**) Total training times. During the first 2 weeks of training, daily training times are reported. After the second week of training up to the end of the experiment (8 weeks post-HF induction) data is reported on a weekly basis. Note that total training time was 70% lower in HF + EX-inT rats. (**C**) Prevalence of tolerant vs intolerant HF rats. (**D**) representative recordings of echocardiography in M-mode from one sedentary HF rat (HF + Sed), one HF + EX-T rat and one HF + EX-inT rat. (**E**–**J**) Summary data of left ventricular (LV) end diastolic diameter (LV_EDD_), LV end systolic diameter (LV_ESD_), LV fractional shortening (LV_FS_), LV end diastolic volume (LV_EDV_), LV end systolic volume (LV_ESV_) and LV ejection fraction (LV_EF_), respectively. Note that LV_FS_, LV_ESV_ and LV_EF_, were reduced, increased and reduced, respectively in HF + EX-T and HF + EX-inT rats compared to HF + Sed rats. *p < 0.05 vs. HF + Sed. HF + Sed, n = 6; HF + EX-T, n = 7; HF + EX-inT, n = 5 rats.

The timeline of the experiments is shown in Fig. 1A. Training times were significantly lower in HF + EX-inT animals, compared to HF + EX-T rats (29.2 ± 10.1 vs. 100.0 ± 13.1% change, HF + EX-inT vs. HF + EX-T, respectively) (Fig. 1B). EX tolerance versus intolerance in HF rats was 61% (n = 17) and 39% (n = 11), respectively (Fig. 1C). In addition, EX tolerance/intolerance was not related to the initial degree of cardiac dysfunction since all groups exhibited similar cardiac dimensions. Indeed, before the beginning of the protocol (2 weeks post-HF surgery) no statistical differences in left ventricle end-diastolic diameter (LV_EDD_) (7.0 ± 0.3 vs. 6.7 ± 1.1 vs. 8.1 ± 0.2 mm, p = 0.54), LV end-systolic diameter (LV_ESD_) (3.8 ± 0.4 vs. 3.3 ± 0.5 vs. 3.8 ± 0.2 mm, p = 0.65), LV_ED_ volume (LV_EDV_) (281.9 ± 21.9 vs. 355.7 ± 24.7 vs. 342.0 ± 19.7 µl, p = 0.20), LV_ESV_ (67.7 ± 9.9 vs. 64.2 ± 9.2 vs. 68.9 ± 10.4 µl, p = 0.54), LV ejection fraction (LV_EF_) (75.3 ± 4.4 vs. 79.8 ± 2.8 vs. 82.3 ± 1.5%, p = 0.38) or LV fractional shortening (LV_FS_) (45.6 ± 3.9 vs. 50.5 ± 3.1 vs. 53.1 ± 1.8%, p = 0.39) (HF + Sed vs. HF + EX-T vs. HF + EX-inT, respectively) were noted between groups.

### Hemodynamic and respiratory measurements in HF animals

Resting physiological parameters at 8 weeks post-HF surgery are displayed in Table 1 and Fig. 1. HF + EX tolerant rats showed a significant increase of LV_ESD_ (4.7 ± 0.4 vs. 3.6 ± 0.3 mm, HF + EX-T vs. HF + Sed rats, respectively, p = 0.007) (Fig. 1D,F) and LV_ESV_ (110.9 ± 18.6 vs. 60.6 ± 13.6 µl, HF + EX-T vs. HF + Sed rats, respectively, p = 0.009), compared to HF + Sed rats (Fig. 1I). In addition, LV_FS_ was significantly decreased in HF + EX-T and HF + EX-inT animals compared to HF + Sed rats (44.5 ± 2.9 and 45.1 ± 4.1 [p = 0.003] vs. 55.2 ± 2.5% [p = 0.002], HF + EX-T and HF + EX-inT vs. HF + Sed rats, respectively) (Fig. 1G), while LV_EF_ was significantly different between HF + EX-T vs. HF + Sed rats (73.3 ± 2.8 vs. 83.9 ± 2.3%, HF + EX-T vs. HF + Sed rats, respectively, p = 0.001) (Fig. 1J). HF + EX-T and HF + EX-inT rats showed no significant differences in LV_EDD_ (p = 0.84), LV_ESD_ (p = 0.69), LV_EDV_ (p = 0.85), LV_ESV_ (p = 0.38), LV_FS_ (p = 0.43) and LV_EF_ (p = 0.38) (Fig. 1). No significant differences in LV_EDD_ and LV_EDV_ were found between groups (Fig. 1E,H, respectively).

**Table 1:** Resting characteristics and cardiovascular parameters in HF after 6 weeks of exercise training (EX).

	HF + Sed (n = 6)	HF + EX-T (n = 7)	HF + EX-inT (n = 5)
**Training effectiveness**			
Soleus/BW (% change)	100.0 ± 6.7	118.1 ± 4.2*	111.0 ± 2.1
**Cardiac hypertrophy**			
HW/BW (mg/g)	3.7 ± 0.3	3.5 ± 0.2	4.4 ± 0.3^+^
**Pulmonary congestion**			
Lung W/D (g/g)	4.2 ± 0.1	4.5 ± 0.1	4.7 ± 0.3
**Blood pressure and HR**			
SBP (mmHg)	101.4 ± 5.3	108.9 ± 6.4	96.0 ± 6.2
DBP (mmHg)	63.3 ± 5.7	72.5 ± 6.0	57.1 ± 3.9
PP (mmHg)	38.1 ± 1.2	36.4 ± 2.2	35.3 ± 7.7
HR (bpm)	318.3 ± 16.2	359.0 ± 14.1	394.3 ± 21.2

Values are expressed as mean ± SEM.

T: tolerant; inT: intolerant; HW: heart weight; HW/BW: heart weight/body weight; lung W/D: Lung Wet/Dry; SBP: systolic blood pressure; DPB: diastolic blood pressure; PP: pulse pressure; HR: heart rate.

One-way ANOVA, Sidak post hoc analysis. *vs. HF + Sed, ^+^vs. HF + EX-T, p < 0.05.

HF + EX-inT rats showed a significant increase of cardiac hypertrophy compared to HF + EX-T animals (Table 1, p = 0.03). HF + EX-T showed an increase in the soleus muscle-to-body weight (soleus/BW) ratio compared to HF + Sed animals (p < 0.05) (Table 1, p = 0.02). HF-EX-inT rats showed a slight but not significant increase in soleus/BW compared to HF + Sed animals (Table 1, p = 0.15). EX did not change cardiac hypertrophy in HF + EX-T animals compared to HF + Sed rats (p = 0.58) (Table 1).

Resting respiratory parameters (in normoxia) are shown in Table 2. No significant changes were found in V_T_ amplitude or R_f_. Accordingly, no changes in respiratory cycle duration or peak flows were found between groups (Table 2).

**Table 2:** Resting ventilatory parameters during normoxia (F_i_O_2_ 21%), hypoxia (F_i_O_2_ 10%), and hypercapnia (F_i_CO_2_ 7%) in HF after 6 weeks of exercise training (EX).

	HF + Sed (N = 5)	HF + EX-T (N = 5)	HF + EX-inT (N = 5)
**Normoxia**			
V_T_ (ml/100 g)	0.27 ± 0.01	0.24 ± 0.04	0.22 ± 0.04
R_f_ (breaths/min)	83.29 ± 5.18	87.89 ± 12.60	88.75 ± 9.96
Ve (mL/min 100 g)	22.62 ± 2.30	21.18 ± 0.83	19.59 ± 2.20
T_i_ (ms)	278.10 ± 53.21	276.90 ± 44.40	245.60 ± 15.46
T_e_ (ms)	420.40 ± 23.48	420.60 ± 138.60	438.70 ± 73.37
T_tot_ (ms)	698.40 ± 54.26	697.40 ± 103.70	684.20 ± 67.07
PiF (ml/s)	9.69 ± 3.34	8.08 ± 2.32	8.38 ± 1.58
PeF (ml/s)	5.755 ± 1.56	5.33 ± 1.54	4.51 ± 0.95
**Hypercapnia**			
V_T_(ml/100 g)	0.37 ± 0.08	0.32 ± 0.02	0.30 ± 0.03*
R_f_ (breaths/min)	201.8 ± 47.99	161.50 ± 16.68*	181.20 ± 29.69
Ve (mL/min 100 g)	74.74 ± 12.98	51.57 ± 3.98*	55.28 ± 4.07*
T_i_ (ms)	155.70 ± 23.01	180.10 ± 18.12*	204.40 ± 16.02*
T_e_ (ms)	177.50 ± 55.20	197.70 ± 28.70	172.30 ± 15.67
T_tot_ (ms)	333.10 ± 97.63	377.90 ± 36.83	376.70 ± 28.69
PiF (ml/s)	18.66 ± 2.80	14.18 ± 1.63*	12.49 ± 2.78*
PeF (ml/s)	15.97 ± 3.09	12.57 ± 2.21*	10.03 ± 2.21*
**Hypoxia**			
V_T_ (ml/100 g)	0.32 ± 0.03	0.35 ± 0.04	0.27 ± 0.07^+^
R_f_ (breaths/min)	193.20 ± 21.31	174.20 ± 14.82	144.70 ± 16.06*^+^
Ve (mL/min 100 g)	61.62 ± 4.34	60.61 ± 4.05	40.86 ± 14.14*^+^
T_i_ (ms)	149.40 ± 13.37	151.30 ± 16.37	194.20 ± 19.57*
T_e_ (ms)	170.50 ± 21.66	184.40 ± 17.90	227.80 ± 31.26*^+^
T_tot_ (ms)	320.00 ± 33.30	335.70 ± 21.52	422.00 ± 46.40*^+^
PiF (ml/s)	18.31 ± 2.82	17.33 ± 2.37	10.17 ± 2.41*^+^
PeF (ml/s)	17.5 ± 2.38	16.17 ± 2.23	11.69 ± 2.97*^+^

Values are expressed as mean ± SEM.

T: tolerant; inT: intolerant; VT: tidal volume; Rf: respiratory frequency; Ve: minute ventilation; Ti: inspiratory time; Te: expiratory time; Ttot: total respiratory time; PiF: peak inspiratory flow; and PeF: peak expiratory flow.

One-way ANOVA, Sidak post hoc analysis. *vs. HF + Sed, ^+^vs. HF + EX-T, p < 0.05.

Cardiac parameters including SV (p = 0.28), SW (p = 0.73), LVESP (p = 0.92), dP/dt_max_ (p = 0.82), dP/dt_min_ (p = 0.86), dV/dt_max_ (p = 0.62), dV/dt_min_ (p = 0.36), TauW (p = 0.48) were not different between groups (Table 3).

**Table 3:** Resting cardiac parameters in HF after 6 weeks of exercise training (EX).

	HF + Sed (N = 6)	HF + EX-T (N = 7)	HF + EX-inT (N = 5)
SV (μl)	293.2 ± 9.4	266.5 ± 31.1	262.8 ± 27.1
CO (μl min)	109,700.0 ± 16,219.1	96,009.1 ± 11,892.0	104,896.0 ± 16,833.3
SW (mmHg μl)	28,285.0 ± 4,253.4	25,433.0 ± 2,549.1	24,298.1 ± 3845.0
LVESP (mmHg)	89.2 ± 6.1	91.2 ± 4.5	88.7 ± 2.4
dP/dt_max_ (mmHg/s)	8288.0 ± 976.5	8945.0 ± 988.8	9063.2 ± 728.2
dP/dt_min_ (mmHg/s)	− 6049.0 ± 852.4	− 6442.4 ± 502.1	− 5966.0 ± 634.9
dV/dt_max_ (μl/s)	15,590.0 ± 2088.0	14,314.1 ± 741.9	16,613.0 ± 2028.3
dV/dt_min_ (μl/s)	− 21,557.0 ± 3039.0	− 26,673.1 ± 2187.0	− 25,177.0 ± 2555.0
TauW (ms)	9.2 ± 0.7	8.1 ± 0.8	7.8 ± 1.0

Values are expressed as mean ± SEM.

T: tolerant; inT: intolerant; SV: stroke volume; SW: Stroke work; LVESP: left ventricle end-systolic pressure; dP/dt_max_: first derivative of maximum intraventricular pressure; dP/dt_min_: first derivative of minimum intraventricular pressure; dV/dt_max_: first derivative of maximum intraventricular volume; dV/dt_min_: first derivative of minimum intraventricular volume; TauW: time constant of relaxation of Weiss.

One-way ANOVA, Sidak post hoc analysis.

### Cardiac sympathetic tone and arrhythmia incidence

**Figure 2:**
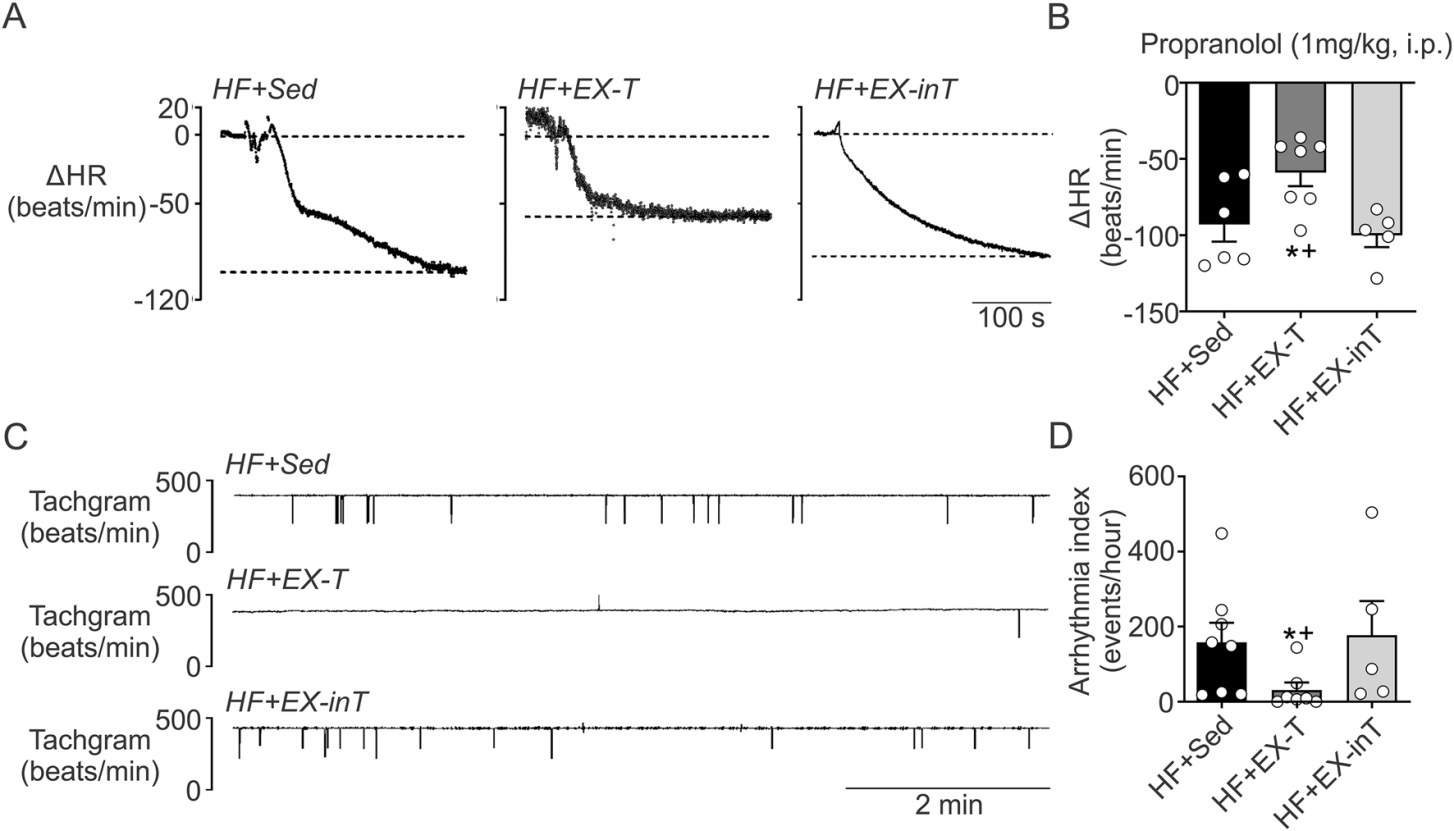
Heart failure rats with exercise training intolerance (HF + EX-inT) displayed similar sympathetic tone and spontaneous arrhythmia incidence compared to sedentary heart failure rats (HF + Sed). (**A**) Representative recordings of heart rate response to propranolol (1 mg/kg i.p.) from one HF + Sed rat, one HF + EX-T rat, and one HF + EX-inT rat. Note that EX did not improve cardiac autonomic control in HF + EX-inT rats. (**B**) summary of the effects of EX on heart rate responses to propranolol. (**C**) representative tachograms from one HF + Sed rat, one HF + EX-T rat, and one HF + EX-inT rat. No beneficial effects of EX on incidence of spontaneous cardiac arrhythmias were found in HF + EX-inT rats. (**D**) Summary of the effects of EX on arrhythmia incidence. *p < 0.05 vs. HF + Sed; ^+^p < 0.05 vs. HF + EX-inT. HF + Sed, n = 6; HF + EX-T, n = 7; HF + EX-inT, n = 5 rats.

The maximum bradycardic response to propranolol was used to estimate cardiac sympathetic tone. HF + EX-T rats had a smaller bradycardic response to propranolol than HF + Sed and HF + EX-inT rats (− 59.0 ± 8.8 vs. − 92.9 ± 11.3 [p = 0.02] and − 99.9 ± 9.8 ΔHR [p = 0.001], HF + EX-T vs. HF + EX-inT and HF + Sed). In contrast, HF + EX-inT rats showed a similar maximum bradycardic response to propranolol compared to HF + Sed rats (− 99.9 ± 9.8 vs. 92.9 ± 11.3 ΔHR, HF + EX-inT vs. HF + Sed rats, respectively, p = 0.64) (Fig. 2A,B).

Arrhythmia incidence was significantly lower in HF + EX-T rats (31.4 ± 19.8 vs. 158.0 ± 51.8 events/hour, HF + EX-T vs. HF + Sed, p = 0.04). HF + EX-inT rats showed no change in arrhythmia incidence compared to HF + Sed animals (Fig. 2C,D).

### Cardiac hemodynamic function

**Figure 3:**
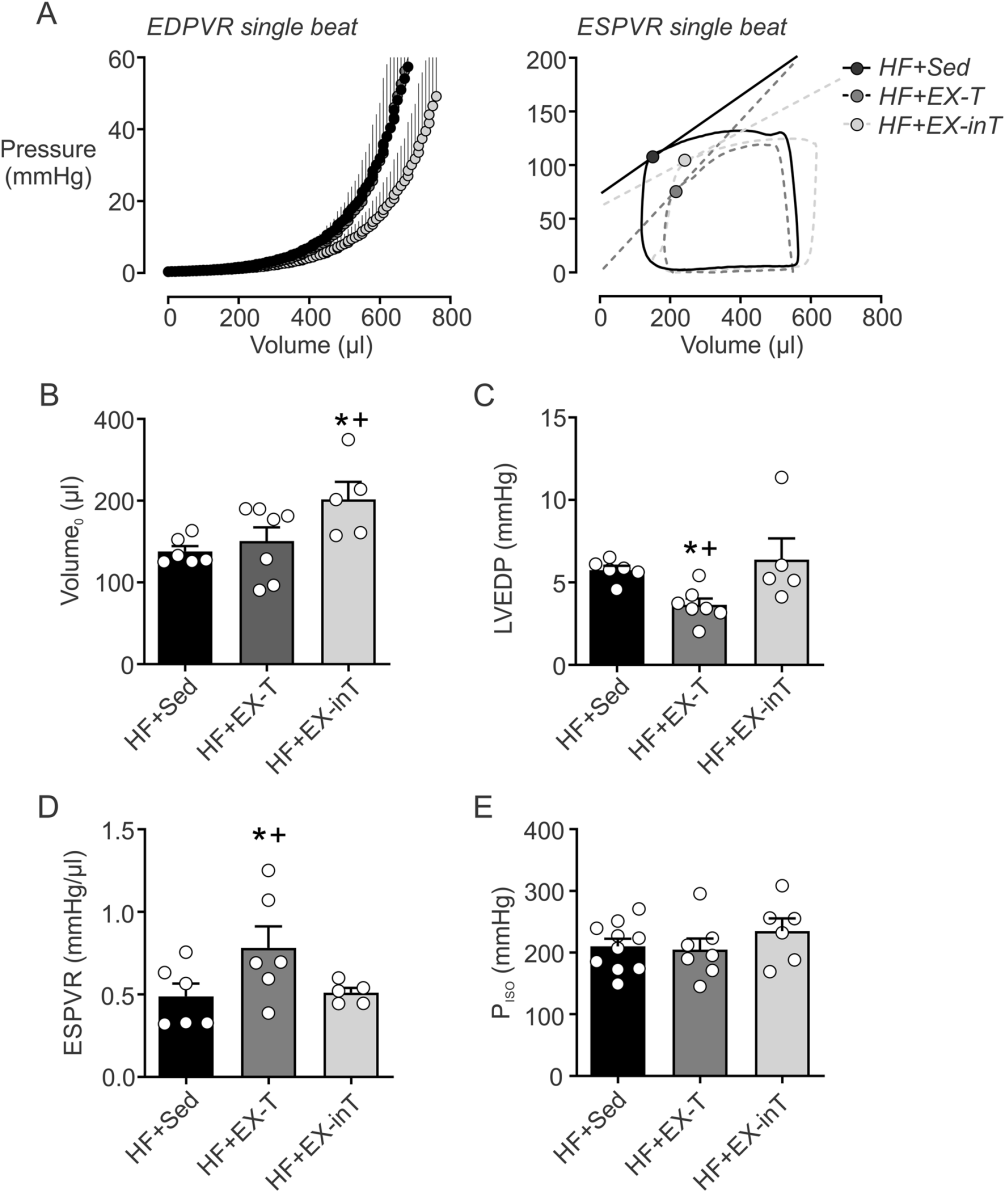
Diastolic function, but not systolic function is worse in heart failure rats with exercise training intolerance (HF + EX-inT). (**A**) (Left) end-diastolic pressure–volume relationship (EDPVR) by single beat analysis and (right) end-systolic pressure–volume relationship (ESPVR) by single beat analysis. (**B**) Summary of the effects of EX on the volume at pressure 0 (EDPVR single beat). Note that HF + EX-inT rats showed a significant increase of volume at pressure 0. (**C**) Summary of the effects of EX on LVEDP. Note that HF + EX-T improves diastolic function. (**D**) Summary of the effects of EX on ESPVR. Importantly, HF + EX-T rats displayed a significant improvement of systolic function. (E) isovolumetric pressure (P_ISO_) was not different between all groups. *p < 0.05 vs. HF + Sed; ^+^p < 0.05 vs. HF + EX-T condition. HF + Sed, n = 6; HF + EX-T, n = 7; HF + EX-inT, n = 5 rats.

Cardiac function parameters are shown in Fig. 3. HF + EX-T animals showed no significant differences in diastolic function compared to HF + Sed rats (Fig. 3A left and B). However, HF + EX-inT had worse diastolic function compared to HF + EX-T and HF + Sed group (V_0_: 289.5 ± 21.3 vs. 254.2 ± 19.4 and 237.8 ± 6.6 µl, HF + EX-inT vs. HF + EX-T and HF + Sed rats, respectively) (Fig. 3A left and B). LVEDP and ESPVR were improved in HF + EX-T rats compared to HF + Sed and HF + EX-inT animals (LVEDP: 3.6 ± 0.3 vs. 5.6 ± 0.2 [p = 0.01] and 6.3 ± 0.8 mmHg [p = 0.01], HF + EX-T vs. HF + Sed and HF + EX-inT, respectively) (ESPVR: 0.8 ± 0.1 vs. 0.5 ± 0.1 [p = 0.03] and 0.5 ± 0.1 mmHg/µl [p = 0.04], HF + EX-T vs. HF + Sed and HF + EX-inT rats, respectively) (Fig. 3C,D). Systolic function in HF + EX-inT rats was not different from HF + Sed rats (Fig. 3A right and D). No differences in maximum isovolumetric pressures were found between groups (Fig. 3E).

### Cardiac responses to chemoreflex activation

**Figure 4:**
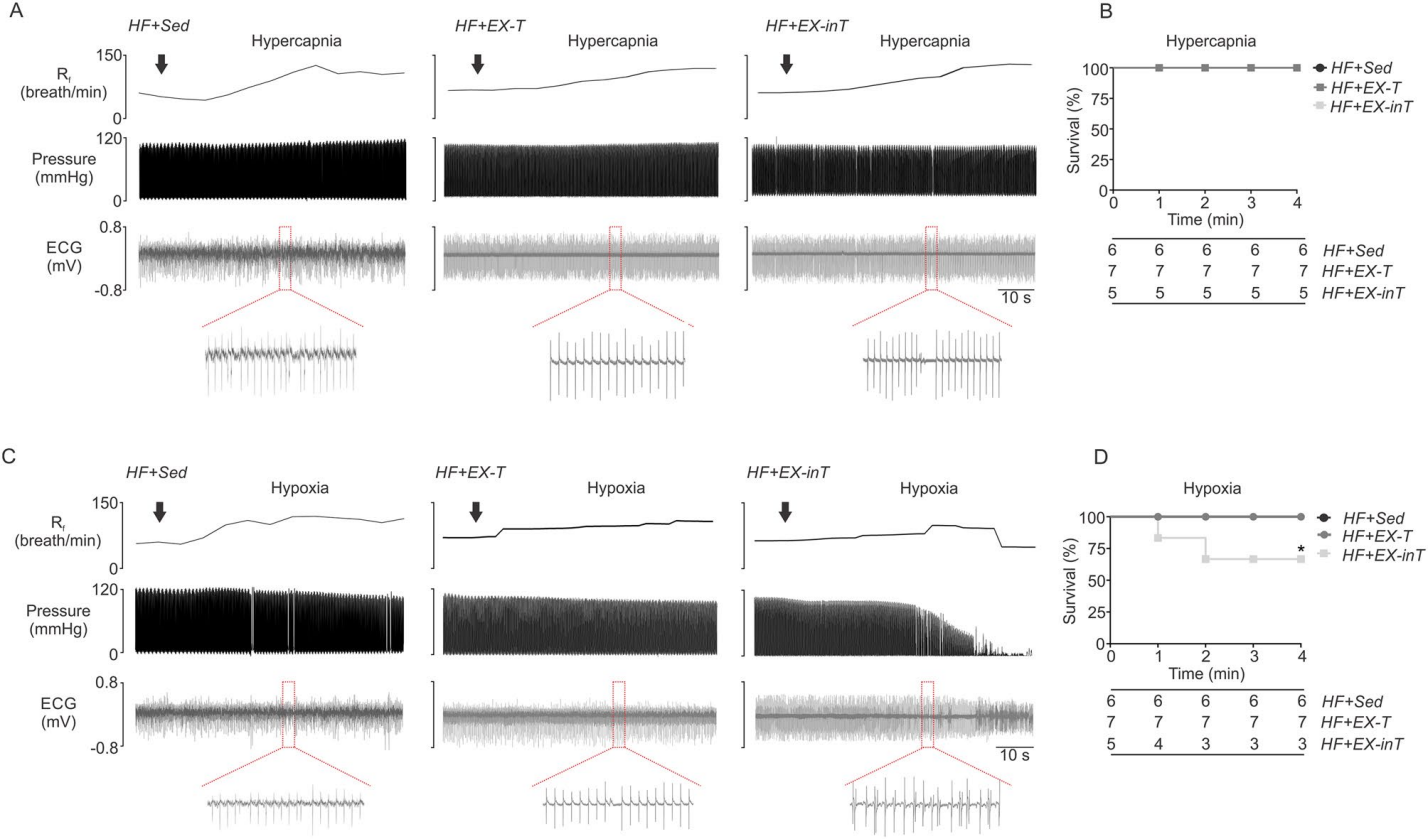
Moderate hypoxia but not hypercapnia increases mortality in exercise intolerant heart failure rats. (**A**) Representative recordings of respiratory frequency (R_f_), intraventricular pressure, and the electrocardiogram (ECG) from one HF + Sed, one HF + EX-T, and one HF + EX-inT rat during acute hypercapnic exposure (F_i_CO_2_ 7%) (arrow, hypercapnia ON). Note that hypercapnic exposure lead to cardiac arrhythmias only in HF + EX-inT. (**B**) Kaplan–Meier survival curves showing no mortality during hypercapnia. (**C**) Representative recording of R_f_, intraventricular pressure, and ECG from one HF + Sed, one HF + EX-T, and one HF + EX-inT rat during acute hypoxic exposure (F_i_O_2_ 10%) (arrow, hypoxia ON). Note that hypoxic exposure lead to cardiac arrhythmias only in HF rats with exercise intolerance (HF + EX-inT); (**D**) Kaplan–Meier survival curves showing that 40% of HF + EX-inT rats died during hypoxia. *p < 0.05 vs. HF + EX-T condition.

Our previous work showed that chemoreflex activation increased cardiac arrhythmias and promoted deterioration in cardiac function in volume-overloaded HF rats^8^. Accordingly, we tested the effects of EX on arrhythmia incidence during stimulation of central and peripheral chemoreflexes. Central chemoreflex activation with acute hypercapnia elicits cardiac arrhythmias in HF + Sed and HF + EX-inT rats to a similar extent (14.4 ± 5.5 vs 17.0 ± 3.8 events/10 min, respectively, p = 0.71). Notably, chemoreflex-induced cardiac arrhythmias were blunted in HF + EX-T rats. Indeed, arrhythmia incidence was ~ threefold lower in HF + EX-T animals compared to HF + Sed animals (5.6 ± 2.1 vs. 14.4 ± 5.5 evens/10 min, HF + EX-T vs. HF + Sed, respectively, p = 0.03) (Fig. 4A,B).

Peripheral chemoreflex stimulation with acute hypoxia did not trigger an increase in cardiac arrhythmias in HF + Sed rats nor in HF + EX-T rats (9.8 ± 4.7 events/10 min, HF + EX-T rats) (Fig. 4C). Indeed, during the hypoxic challenge both HF + Sed and HF + EX-T animals displayed an increase R_f_ without changes in intraventricular pressure and/or EKG (Fig. 4C, left panel). In contrast, peripheral chemoreflex stimulation induced a marked increase in cardiac arrhythmias and related mortality in HF + EX-inT rats (Fig. 4C). Within a minute of hypoxic stimulation, arrhythmic events begin to appear and were accompanied by decreases in intraventricular pressures (Fig. 4C, right panel). Peripheral chemoreflex stimulation led to mortality in 40% of HF + EX-inT rats but did not cause mortality in the other experimental groups (Fig. 4D).

### Hypoxic and hypercapnic ventilatory responses

Responses to central and peripheral chemoreflex stimulation were evaluated through the hypercapnic (HCVR: F_i_CO_2_ 7%) and hypoxic (HVR: F_i_O_2_ 10%) ventilatory responses, respectively. HVR and HVCR assessed before the onset of the EX-program (2 weeks post-HF surgery) was not statistically differences between groups (HVR: 2.6 ± 0.3 vs. 3.6 ± 0.3 vs. 2.5 ± 0.4 ΔV_E_/ΔF_i_O_2_% [p = 0.89]; and HCVR: 2.3 ± 0.6 vs. 2.9 ± 0.6 vs. 2.5 ± 0.5 ΔV_E_/ΔF_i_CO_2_% [p = 0.11], HF + Sed vs. HF + EX-T vs. HF + EX-inT, respectively).

**Figure 5:**
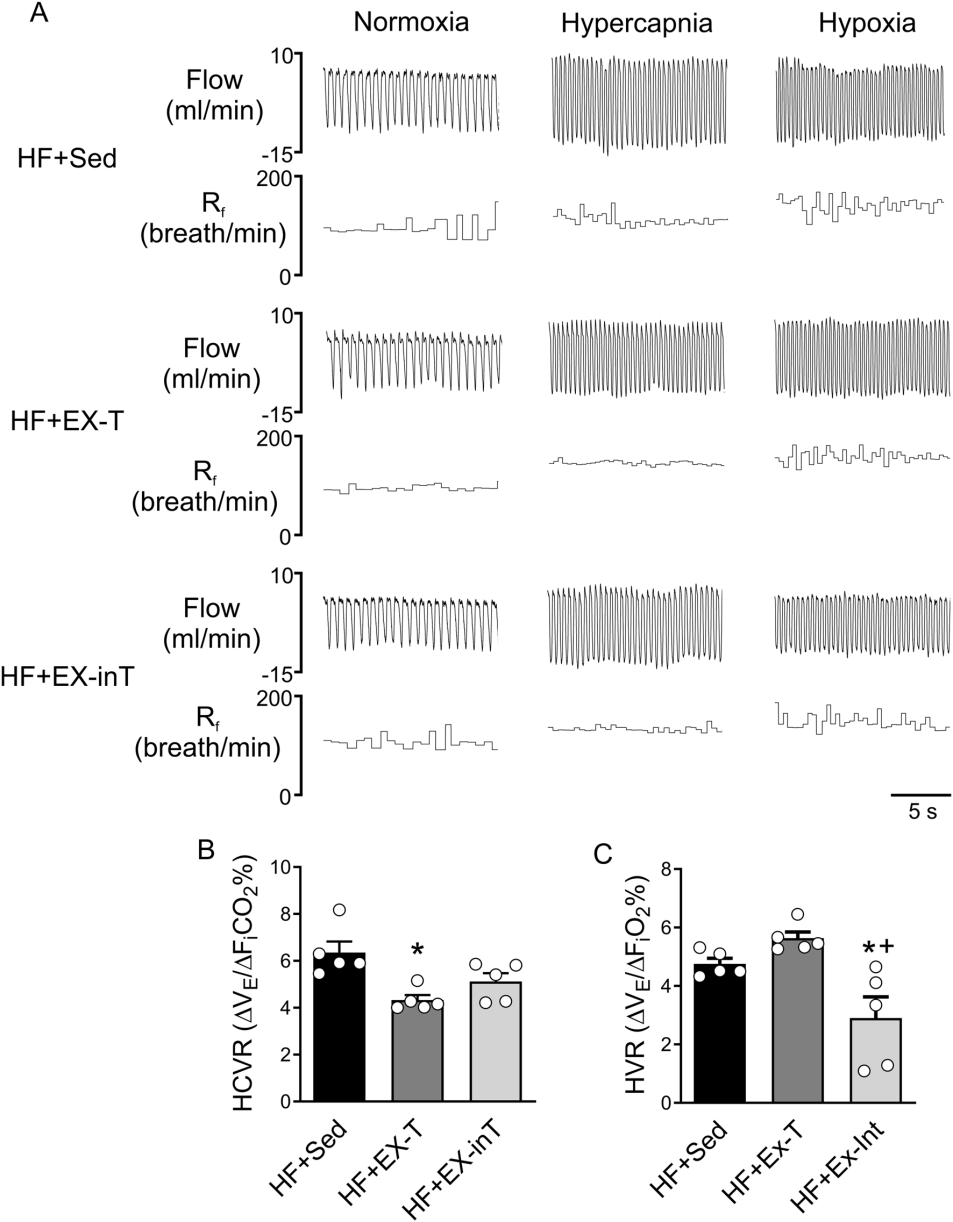
Peripheral chemoreflex gain is lower in exercise intolerant heart failure rats (HF + EX-inT). (**A**) Representative traces of whole-body plethysmography from one HF + Sed rat, one HF + EX-T rat, and one HF + EX-inT rat during normoxia (F_i_O_2_ 21%), hypercapnia (F_i_CO_2_ 7%), and hypoxia (F_i_O_2_ 10%). (**B**) Summary data of hypercapnic ventilatory response HCVR. (**C**) Summary effects of EX on HVR. Note that HF + EX-inT showed a lower hypoxic ventilatory response (HVR) compared to the HF + Sed rats and HF + EX-T rats. *p < 0.05 vs. HF + Sed; ^+^p < 0.05 vs. HF + EX-T. HF + Sed, n = 6; HF + EX-T, n = 6; HF + EX-inT, n = 6.

At 8 weeks post-HF induction, HF + EX-T rats had significantly lower HCVR compared to HF + Sed (3.1 ± 0.8 vs. 6.4 ± 0.4 ΔV_E_/ΔF_i_CO_2_%, HF + EX-T vs. HF + Sed, respectively, p = 0.04) (Fig. 5A,B), but HCVR was not different compared to HF + EX-inT animals (p = 0.42) (Fig. 5B). In contrast, HCVR in HF + EX-inT was similar to that in HF + Sed (p = 0.08) (Fig. 5B).

HVR in HF + EX-T was not significantly different compared to HF + Sed rats (Fig. 5A,C). While HF + EX-inT rats showed a significantly lower HVR than HF + EX-T and HF + Sed animals (2.9 ± 0.7 vs. 5.6 ± 0.2 [p = 0.003] and 4.8 ± 0.2 ΔV_E_/ΔF_i_O_2_% [p = 0.03], HF + EX-inT vs. HF + EX-T and HF + Sed) (Fig. 5A,C). In addition, HF + EX-inT animals showed significant differences in respiratory frequency (R_f_), minute ventilation (V_e_), expiratory time (T_e_), total respiratory time (T_tot_), peak inspiratory flow (PiF) and peak expiratory flow (PeF) responses to hypoxia compared to HF + EX-T and HF + Sed animals (Fig. 5 and Table 2). V_T_ was significantly lower in HF + EX-inT rats compared to the HF + EX-T animals (Table 2). In addition, inspiratory time (Ti) was significantly higher in HF + EX-inT rats compared to HF + Sed animals (Table 2).

### Carotid body ablation (CBA) and EX tolerance in HF rats

**Figure 6:**
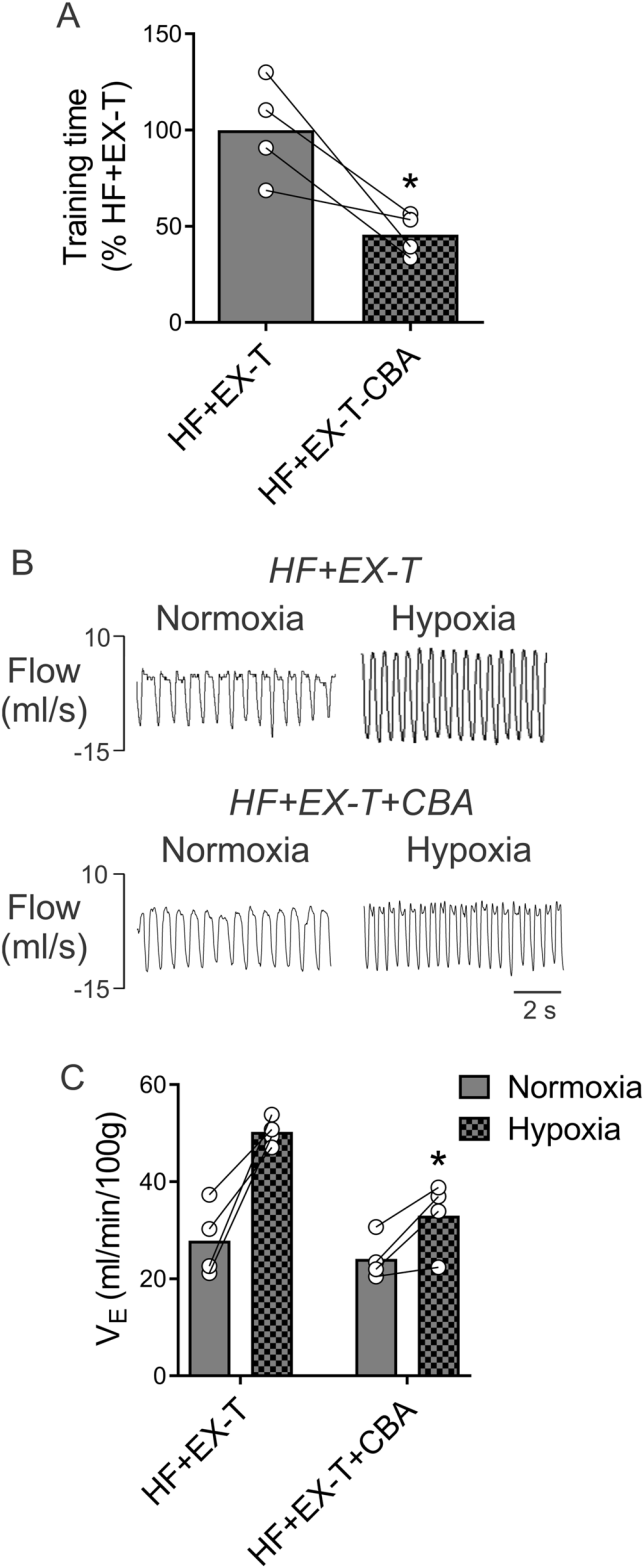
Carotid body ablation (CBA) in HF-EX-T rats induces a phenotypic switch from exercise tolerant to exercise intolerant. (**A**) Summary data showing training time before and after CBA in HF-EX-T rats. Note that CBA was associated with significantly lower training time in rats that were previously tolerant of EX. (**B**) Representative traces of HVR from one HF + EX-T rat before and 2 weeks post-CBA. (**C**) Summary data of minute ventilation during normoxia and hypoxia. Note that ventilatory response to hypoxia was significantly reduced after CBA. *p < 0.05 vs. HF + EX-T during hypoxia. HF + EX-T, n = 4; HF + EX-T + CBA, n = 4.

The effects of CBA on training times are displayed in Fig. 6. Total training time was reduced by ~ 50% in HF + EX-T rats after CBA compared to previous EX times obtained before CBA (Fig. 6A). CBA resulted in a significant reduction in the ventilatory response to hypoxia in HF + EX-T (50.3 ± 1.4 vs. 33.1 ± 3.7 ml/min/100 g, before vs. after CBA in HF + EX-T; Fig. 6B,C, p = 0.005).

## Discussion

In the present study we found that: (i) EX-inT was present in 39% of high-output HF rats; (ii) EX-inT was not related to the initial degree of cardiac failure nor to HCVR/HVR prior to the onset of EX in HF rats; (iii) HF + EX-inT rats had similar degrees of autonomic dysfunction, arrhythmia incidence, and cardiac systolic dysfunction compared to HF sedentary rats; (iv) EX-inT results in a greater degree of cardiac diastolic dysfunction in HF; (v) lower HVR in HF + EX-inT was associated with increased incidence of cardiac arrhythmias and higher mortality during hypoxic challenge; and (vi) ablation of peripheral chemoreceptors in HF + EX-T rats was sufficient to induce a phenotype similar to EX-inT animals. Taken together, our data suggest that decreased peripheral chemoreflex gain contributes to EX-inT in HF. Based on our findings it is plausible that reductions in chemoreflex gain in a subset of HF patients may lead to EX-inT, further abnormalities in cardiac function, and potentially predispose to severe arrhythmogenesis and increased mortality risk during moderate hypoxia exposure.

Intolerance to exercise is a well-recognized characteristic of HF^22^. HF patients typically have reduced exercise and functional capacity and experience dyspnea during daily activities^21,27^. Interestingly, intolerance to physical exercise is observed in both types of HF (i.e. reduced and preserved ejection fraction)^27^. Current theories on exercise intolerance center on an inability to adequately increase cardiac output during exercise and reductions in muscle blood flow secondary to reduced EF and potentially abnormalities in muscle metabolism^20^. In addition to cardiac, vascular, and metabolic responses to exercise, respiratory adjustments to exercise are just as crucial to maintaining homeostasis and therefore exercise tolerance.

In the present study, we used a volume-overload HF model to study the contribution of chemoreflexes to exercise tolerance in the absence of reduced EF^5,26^. We showed that volume-overload HF rats display exercise intolerance independent of the initial degree of cardiac failure. With respect to respiratory control, we observed that these animals displayed decreased HVR after training despite the fact that HVR was similar between all groups prior to the onset of the EX-program. Therefore, it is reasonable to hypothesize that exercise intolerance in volume-overload HF stems in part from a reduction in peripheral chemoreflex gain. To test this assumption, we eliminated the peripheral chemoreflex in HF rats that were previously proven to be exercise tolerant. These HF + EX-T rats underwent bilateral ablation of the carotid bodies after 6 weeks of EX. Notably, we found that HF + EX-T rats showed a significant loss in EX performance at 2 weeks post-ablation. Indeed, total training times in HF + EX-T rats that underwent ablation were not significantly different from the values obtained in HF + EX-inT rats. This result strongly supports a role for peripheral chemoreceptors in contributing to exercise tolerance in HF. Based on our findings, the precise mechanisms by which peripheral chemoreceptors contribute to exercise tolerance in volume-overload HF, a model of HF with no reductions in EF, warrants further investigation.

Impairment of systolic and diastolic function has been observed in patients with HF and in animal models of HF^1,3,8,28^. Whether or not EX results in improvements in diastolic function in HF is still controversial. A meta-analysis performed by Pandey et al.^29^ showed that preserved ejection fraction HF patients did not experience significant improvements in diastolic function following EX^29^, and our previous work showing that EX training did not improve diastolic dysfunction in high-output HF rats^1^ is consistent with these findings. The results of the present study in HF + EX-T rats confirm and extend our previous observations by illustrating the detrimental effects of EX in EX-inT HF rats. We found that EX intolerance in HF rats was associated with worse diastolic function after completing an EX protocol. This result suggests that in some HF populations with no compromise in ejection fraction physical exercise may be detrimental. Interestingly, this concern has previously been discussed as an important factor in disease progression and decompensation in human HF independent of its etiology^20^.

Similar to what is known about the effects of EX on diastolic function in HF, evidence regarding the beneficial effects of EX on systolic function is limited and controversial^1,15,30,31^. It has been shown that endurance EX in HF patients results in minor improvements in systolic function^32^, however in these studies no effort was made to distinguish between EX tolerant vs. intolerant patients^32^. In contrast, we previously reported that EX improves systolic function in HF rats^1^. It is worth noting that in the present study we found similar beneficial effects of EX on cardiac contractility, but that this was restricted to EX tolerant HF animals. Indeed, no beneficial effects of EX on cardiac contractility were observed in HF + EX-inT rats. For future studies to accurately assess the beneficial effects of EX on cardiac function in HF it is important to identify and differentiate the proportion of the patient population that do not tolerate EX.

Autonomic dysfunction characterized by sympatho-vagal imbalance is a hallmark of HF in humans and is faithfully reproduced in animal models of HF^1,8,33^. Our previous work and that of others has shown that volume-overload HF rats exhibit autonomic imbalance characterized by heightened sympathetic activity^1,3–5,8,34^. It has been proposed that autonomic imbalance, mainly sympathoexcitation, contributes to EX intolerance in HF by reducing muscle blood flow along with increases in peripheral vascular resistance^23^. Importantly, volume-overload HF with preserved ejection fraction is a particularly useful model for examining the effects of EX intolerance in the absence of overt reductions in cardiac output. We found that EX was an effective means to reduce cardiac sympathetic tone in HF rats that tolerate EX. This is in agreement with our previous observations^1^; but, this research also extends our previous findings by showing that EX has no salutary effect on sympathetic activity in HF rats with EX intolerance. In this study, cardiac chronotropic responses to propranolol from HF + EX-inT rats were undistinguishable from those in in sedentary HF rats. Whether this lasting heightened sympathetic activity in HF + EX-inT group contributes to the EX intolerant phenotype in HF requires further study.

Cardiac arrhythmias are a major contributor to mortality in patients with HF^29^ and are often associated with increased cardiac sympathetic tone^4,35^. In our previous studies we showed that volume-overload HF rats had a higher incidence of cardiac arrhythmias than healthy control rats and that EX training resulted in significantly lower occurrence of these arrhythmias^1,8^. In this study we extend our previous findings with the observation that EX results in no beneficial effect on cardiac arrhythmias in HF rats with EX intolerance. It is important to note that HF + EX-inT rats do not experience improvements in cardiac sympathetic tone as observed in HF rats that tolerate EX. Previously, we reported that cardiac arrhythmias in volume-overload HF rats are driven primarily by sympathetic activation^3^, therefore it is plausible that the lack of effects of EX on cardiac arrhythmias in HF + EX-inT rats was related, at least in part, to the inability of EX to reduce sympathoexcitation. However, we cannot rule out the possibility that cardiac remodeling occurred following EX training in HF + EX-inT rats which contributed to an arrhythmic substrate.

It has been proposed that chemoreflex-mediated sympathoexcitation is a major contributor to the progression of HF independent of its etiology^4,6,35^. Previously we found that volume-overload HF is associated with enhanced central chemoreflex gain and autonomic dysfunction, but that these animals did not exhibit changes in peripheral chemoreflex gain^8^. In the present study we showed that EX in HF + EX-T rats significantly reduced central chemoreflex gain without changing peripheral chemoreflex gain. Therefore, it is possible that reductions in central chemoreflex gain in HF + EX-T rats following EX contribute to the improvements we observed in autonomic and cardiac function. In contrast, EX in HF + EX-inT rats had no discernable effect on central chemoreflex gain compared to HF sedentary rats. Surprisingly, we found that peripheral chemoreflex gain was attenuated after EX in HF + EX-inT rats. This finding is of note in part because when exposed to moderate hypoxia, HF + EX-inT rats had increased incidence of cardiac arrhythmias and higher mortality compared to HF + Sed animals. It is plausible that a diminished hypoxic ventilatory response compromises cardiac oxygen supply during hypoxic challenge in HF + EX-inT animals triggering lethal cardiac arrhythmias.

Whether a decrease in peripheral chemoreflex gain affects tolerance to EX in HF has not been previously addressed. It has been shown that CB ablation in humans results in a significant reduction of exercise hyperpnea^36^, but the extent to which this affects exercise tolerance is unclear. It has been proposed that carotid body chemoreceptors act not only to maintain arterial blood gas homeostasis, but that they also serve an important role as metabolic sensors. Recently, Chang et al.^36^, showed that carotid bodies respond to increases in extracellular lactate and can elicit a ventilatory response to physiologically relevant lactate concentrations. It is widely known that plasma lactate levels increase during acute exercise (of sufficient intensity), therefore, it is possible that peripheral chemoreceptors modulate the ventilatory adjustments and tolerance to EX in HF. In the case of a decrease in peripheral chemoreflex gain (as we observed) during higher intensity exercise, a loss in the sensitivity to metabolic by-products by peripheral chemoreceptors would adversely affect ventilatory responses to EX and potentially contribute to EX intolerance.

Since our HF + EX-inT animals displayed decreased peripheral chemoreflex gain we decided to determine if reducing peripheral chemoreflex gain (via carotid body ablation) would produce a phenotype switch in HF animals that had previously demonstrated good tolerance for EX. Notably, ablation of the carotid bodies in HF + EX-T rats did indeed result in a phenotype switch, transforming the exercise tolerant animal into an intolerant one. Taken together, our observations suggest that reductions in peripheral chemoreflex gain may play an important role in determining EX tolerance in volume-overload HF. Measuring ventilation during exercise in rodents is very difficult, however future studies that address the precise role of peripheral chemoreceptors in EX ventilation in HF would add important insight to our findings.

In addition to our hypothesis regarding reductions in chemosensitivity, there are certainly other factors which may contribute to EX intolerance in HF. Previous studies have shown right heart to pulmonary circulation uncoupling and ventilatory inefficiency in HF patients with both reduced and preserved ejection fraction. As our study did not directly address this hypothesis, our data do not support or refute this possibility. With that said, our data showing that CB ablation in formerly EX tolerant HF animals results in EX intolerance do lend strong support to the notion that CB plays a role in EX tolerance in some settings. This does not preclude the potential contribution of changes in ventilatory efficiency or uncoupling of right heart and pulmonary circulation. While some studies have looked at the role of the CB in controlling hemodynamic function and EX performance during HF, these were performed in HF patients with reduced EF. Given the distinct changes in chemoreflex function that occur in different HF etiologies (preserved vs. reduced EF) a direct comparison between these studies is difficult. Interestingly, Collins et al.^37^ and Edgell et al.^38^ noted an influence of CB on cardiovascular regulation at rest and during exercise in HFrEF patients but did not find that CB inhibition had any beneficial effect on EX tolerance. The implications of these studies are unclear as the relative chemosensitivity of these patients was not quantified, and not all HFrEF patients have altered chemoreflex function. Furthermore, HFrEF patients with altered chemoreflex function are more likely to have enhanced chemosensitivity/HVR, whereas our findings in a high output model of HF is of reduced HVR. It is possible that a reduced HVR is a product of pulmonary mechanical restraint on ventilation, however we did not observe any evidence of significant changes in pulmonary ventilation between groups or lung wet-to-dry weight ratios. As mentioned previously our studies were not designed to address this question, but they provide no evidence to support it.

## Limitations

It is important to acknowledge the limitations of the chosen experimental model of HF. In this model, as with many other HF models, none of the common comorbidities that are typically associated with development or progression of HF ((i.e. coronary artery disease, diabetes mellitus, atrial fibrillation and hypertension)^33,39^ are present. In this study we used volume overload to create a high-output model of HF, but we feel caution is required when trying to extrapolate our findings to human HF^40,41^. Further validation is required before generalizing our results to the clinical setting. Other limitations of the present study include the use of anesthesia during invasive measurements of cardiac function and autonomic tone. This has the potential to impact our results due to the well-known cardiovascular effects of anesthetics. However, it is worth noting that the exact same anesthetic agent and depth of anesthesia were used in all three experimental conditions. So, any effect of anesthesia on our measurements would be expected to be similar across experimental conditions. Also, in our study we did not assess right ventricular (RV) function. Since alterations in pulmonary vascular mechanics and right heart pressure due to congestion could affect exercise performance, a full study on RV function is needed to determine the extent to which this plays a role in exercise intolerance in this model. We did not find significant changes in lung wet-to-dry weight ratio in EX-inT HF rats, but we cannot rule out the possibility that subtle changes in pulmonary mechanics may have adversely affected RV function. Additionally, our experimental design did not allow for measurement of ventilation or other cardiovascular and metabolic parameters during EX sessions. Furthermore, we did not test whether the animals displayed different peripheral chemoreflex sensitivity prior to HF induction which may, or may not, have influenced EX performance. Furthermore, our study cannot discern whether changes in HVR precede or coincide with EX-inT in volume overload HF. Therefore, future studies probably at early timepoints will be required to clarify the precise moment at which HVR and EX-inT develops in HF condition. Nevertheless, previous studies have shown little interindividual variability in chemoreflex drive in control conditions (before HF)^4,8,35^. Therefore, we consider it highly unlikely that initial differences, if any, in chemoreflex sensitivity influenced our results. Additional studies addressing cardiorespiratory responses before and during acute exercise will provide additional insights into EX intolerance in HF.

## Conclusions

Exercise intolerant HF rats display low peripheral chemoreflex gain and worse cardiac function than exercise tolerant HF rats. Interestingly, ablation of peripheral chemoreceptors in exercise tolerant HF rats resulted in transformation of exercise-tolerant animals to exercise-intolerant animals. Our data suggest that lower peripheral chemoreflex sensitivity contributes, at least in part, to exercise intolerance in volume-overload HF rats.

## Materials and methods

### Ethical approval and animals

Forty male Sprague–Dawley rats (250 ± 20 g) were used in these experiments. All experiments were performed 8-weeks following induction of HF. In accordance with the National Institutes of Health Guide for the Care and Use of Laboratory Animals and the Guía para el Cuidado y Uso de los Animales de Laboratorio from CONICYT, all animals were kept at controlled room temperature under a 12 h light/dark cycle with ad libitum access to food and water. All experimental protocols were approved by the Ethics Committee for Animal Experiments of the Pontificia Universidad Católica de Chile (#170710022) and were performed according to the ARRIVE Guidelines. All experiments were performed in the Laboratory of Cardiorespiratory Control in the Department of Physiology of the Pontificia Universidad Católica de Chile. At the end of the experimental period all animals were humanely euthanized via anesthetic overdose (sodium pentobarbital 100 mg/kg i.p.).

**Figure 7:**
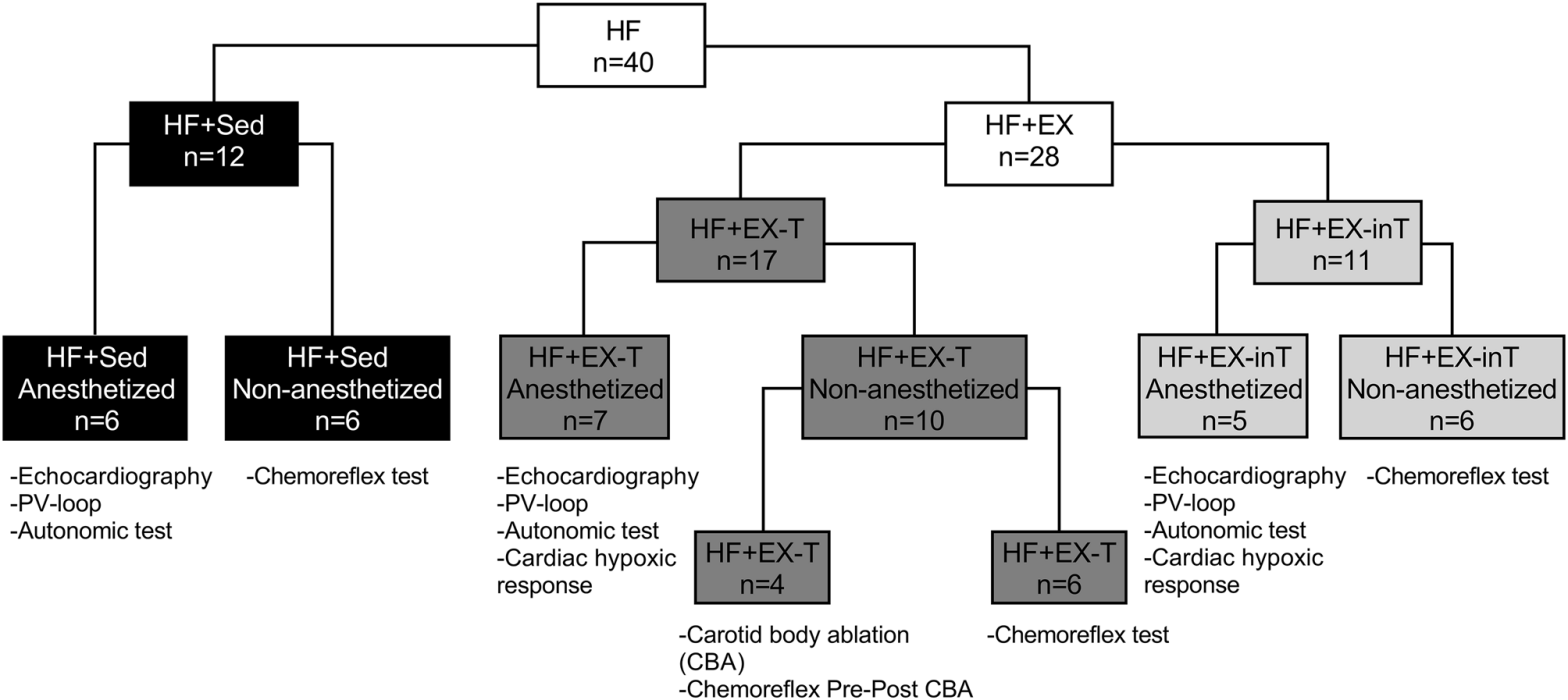
Schematic representation of used animals in the present study. From 40 heart failure (HF) animals, n = 12 were assigned to sedentary (Sed) condition and n = 28 were assigned to exercise training (EX). From Sed animals, n = 6 were allocated to anesthetized experiments and n = 6 to non-anesthetized preparation. From HF + EX animals, n = 17 were classified as HF tolerant (HF + EX-T) animals and n = 11 were classified as HF intolerant (HF + EX-inT) rats. From total HF + EX-T animals, n = 7 were allocated to anesthetized experiments and n = 10 to non-anesthetized preparation (n = 4 to carotid body ablation and n = 6 to chemoreflex test at 8th weeks). From HF + EX-inT rats, n = 5 were allocated to anesthetized experiments and n = 6 to non-anesthetized preparation.

Two-weeks after HF induction surgery, rats were randomly allocated to endurance exercise training (EX; n = 28) or sedentary (Sed; n = 12) conditions. Sedentary animals were assigned to plethysmography experiments (n = 6) and echocardiography and cardiac function experiments (n = 6). Exercise tolerance was evaluated during the 60 min training session duration. Then, based on training times, rats that completed all training sessions (60 min of total training time) were assigned to the HF + EX-T (n = 17) group and those who failed to complete or showed less than 50% of completion of the 60 min session time (n = 11) were classified as HF + EX-inT^42^. From inT animals, n = 6 rats were assigned to plethysmography experiments and n = 5 rats, were allocated to anesthetized preparation at week 8. From tolerant animals, n = 7 rats were allocated to anesthetized experiments, n = 6 rats were assigned to plethysmography experiments. Finally, n = 4 rats from HF + EX-T rats were used for CBA experiments (Fig. 7).

### Volume overload HF model

Volume overload was used to induce HF as described previously^1,3,8,43–45^. Briefly, under anesthesia (2% isoflurane/ 98% O_2_) a laparotomy was performed and an anastomosis was created between abdominal aorta and inferior vena cava vessels using 1.20 × 40 mm needle (BD Precision Glide). The opening in the aorta was closed using tissue adhesive (Histoacryl, Braun). The abdomen was then closed in layers. Post-operative management consisted of administration of 5 mg enrofloxacin (s.c.), 1 mg ketoprofen (s.c.), 5 ml saline solution (i.p.) and 2% lidocaine hydrochloride jelly (topical). Success rates for this surgical approach range from ~ 80 to 90%. The inclusion criteria for allocation to the HF group included: concomitant increases in EDV and SV (~ twofold increase/each), and the presence of preserved EF^1,3,8,43–45^.

### Exercise training protocol

The EX-training protocol was similar to previous studies^1^. Briefly, rats ran on a motor-driven treadmill (PanLab, Harvard Apparatus, USA) at low speed (10 m/min), low % grade (0%), and for a short duration (10 min/day) during the first 2 weeks of the training program. Then, intensity and duration were gradually increased to 25 m/min at 10% grade for 60 min/day until they completed 6 weeks of training. Regardless of whether HF rats were classified as EX-T or EX-inT, all rats exercised for 6 weeks. Exercise intolerance was defined as the inability to complete one full training session (i.e. animals remained in the electrified grid for more than 5 s for 3 consecutive times). Exercise intolerance was assessed 2 weeks after initiation of the exercise program. Soleus to body weight ratio was calculated in each animal to estimate effectiveness of EX as previously described^1^.

### Echocardiography

To assess left ventricular function, echocardiography was performed at 2 weeks post-HF surgery and again at the end of the protocol (8 weeks post HF surgery), as previously described^46^. Echocardiography was repeated in all groups at week 8 post-HF induction after completing EX or Sed protocols. Briefly, under anesthesia (isoflurane 1.5–2.0%, 97% O_2_) rats were placed in supine position and scanned in M-mode with an echocardiograph (Samsung Medison Co., Seoul, South Korea), using a 12-MHz electronic transducer. Images were obtained from the left parasternal short-axis views of the left ventricle (LV) at the level of papillary muscles^47^. Left ventricular end-diastolic diameter (LV_EDD_) and left ventricular end-systolic diameter (LV_ESD_) were measured. Subsequently the left ventricular end-diastolic volume (LV_EDV_), left ventricular end-systolic volume (LV_ESV_), ejection fraction (LV_EF_) and fractional shortening (LV_FS_) were calculated. Animals were classified as high-output HF if they exhibited a minimum of a 2.5-fold increase in SV and LV_EDV_, as previously described^1,3,5,8^.

### Invasive cardiac hemodynamics

Invasive assessment of left ventricular hemodynamic function was performed at the end of the experimental period (8 weeks post HF induction). Rats were anesthetized with α-chloralose (40 mg/kg) and urethane (800 mg/kg), and then were intubated (16-g cannula). After this a 2F pressure–volume (PV) conductance catheter (Millar, SPR-869) was placed into the right carotid artery and advanced into the left ventricle^8,48–50^. In addition, another catheter was inserted in the jugular vein for bolus calibration^48^. Before LV placement of the PV catheter, systolic blood pressure (SBP), diastolic blood pressure (DBP), mean arterial blood pressure (MABP), pulse pressure (PP) and heart rate (HR) were determined. Systolic and diastolic cardiac function were determined by single beat analysis^51–53^. After an equilibration period (25-min), PV loops were recorded, and LV hemodynamic parameters were calculated using 10–15 successive PV loops. PV loop parameters were left ventricular end-systolic pressure (LV_ESP_) and left ventricular end-diastolic pressure (LV_EDP_). Load-dependent cardiac function parameters were determined by means of the calculation of dp/dt_max_ and dp/dt_min_. Load-independent systolic cardiac function was determined by calculating the slope of end-systolic pressure–volume relationship (ESPVR) from one cardiac cycle. Diastolic cardiac function was determined by volume at pressure 0, from one cardiac cycle. In addition, during PV-loop preparation cardiovascular response to hypoxia (F_i_O_2_ 10% O_2_/balance N_2_) and hypercapnia (F_i_CO_2_ 7% CO_2_/93% O_2_) were evaluated. Volumes were calibrated using arterial blood by the cuvette calibration method and NaCl 30% i.v. bolus for determination of parallel conductance^8,44^. Data analysis was performed using the PV loop module of LabChart 8.0 software.

### Cardiac sympathetic tone

We determined cardiac sympathetic tone by measuring the maximum bradycardic response to propranolol, as previously described^3,8^. Briefly, during PV-loop recording a bolus of propranolol was injected (1 mg/kg i.v.) and the maximal chronotropic response was quantified. Sympathetic tone was quantified as the change in heart rate (ΔHR) in response to propranolol.

### Arrhythmia incidence

Arrhythmia incidence was measure as previously described^1,3–5,8^. HR was derived from blood pressure dP/dt waveforms. Irregular heartbeats were visually inspected and counted as previously described^1,3–5,8,54^. Arrhythmias were defined as premature or delayed beats with changes greater than 3 standard deviations (SD) from the mean beat-to-beat interval duration^1,3–5,8^. Arrhythmia incidence was expressed as events/hour.

### Central and peripheral chemoreflex function

Chemoreflex function was assessed at 2 weeks post-HF induction and at the end of the experimental protocol (8 weeks post-HF induction). Central and peripheral chemoreflex sensitivity was assessed by allowing the rats to breathe a mixture of hypercapnic or hypoxic gas, respectively^4,8^. Briefly, unrestrained whole-body plethysmography was used to measure or calculate the following: tidal volume V_T_, respiratory frequency R_f_, minute ventilation (V_E_), inspiratory time (T_i_), expiratory time (T_e_), total respiratory time (T_tot_), peak inspiratory flow (PiF) and peak expiratory flow (PeF). These were measured during stimulation of central chemoreceptors with hyperoxic hypercapnia (7% CO_2_/93% O_2_, for 10 min) and during stimulation of peripheral chemoreceptors with poikilocapnic hypoxia (10% O_2_/balance N_2_, for 10 min). The hypercapnic ventilatory response (HCVR) was obtained by calculating the slope of the linear regression adjustment of the V_E_ response following F_i_CO_2_ 0.03% and 7%, as previously described^8^. The hypoxic ventilatory response (HVR) was obtained by calculating the slope of the linear regression adjustment of the V_E_ response following F_i_O_2_ 21% and 10% challenges, as previously described^8^. All recordings were made at an ambient temperature of 25 ± 2 °C. Data was calculated using ECG auto software (EMKA technologies, France)^4,8^.

### Carotid body ablation (CBA)

After 6 weeks of exercise training the carotid bodies (CBs) were ablated as previously described^4^. Briefly, rats were anesthetized with 2% isoflurane in O_2_. Under sterile surgical conditions, the CBs were exposed via a ventral incision on the neck, visually identified, and cryogenically destroy^4^. The effectiveness of this maneuver was confirmed by the lack of hypoxic ventilatory responses immediately after recovery from surgery^4^. Post-operative management consisted of administration of 5 mg enrofloxacin (s.c.), 1 mg ketoprofen (s.c.), 5 ml saline solution (i.p.) and 2% lidocaine hydrochloride jelly (topical).

### Statistical analysis

Data were expressed as mean ± standard error of the mean (SEM). All data were subjected to Shapiro–Wilk normality test. Differences among groups were assessed using one-way ANOVA, followed by Holm-Sidak post hoc comparisons. An alpha of p < 0.05 was considered statistically significant. All analysis was performed with Prism version 8.4.0 (GraphPad Software, USA).
